# Colorimetric-Based Detection of TNT Explosives Using Functionalized Silica Nanoparticles

**DOI:** 10.3390/s150612891

**Published:** 2015-06-03

**Authors:** Noorhayati Idros, Man Yi Ho, Mike Pivnenko, Malik M. Qasim, Hua Xu, Zhongze Gu, Daping Chu

**Affiliations:** 1 Electrical Engineering Division, Department of Engineering, University of Cambridge, 9 JJ Thomson Avenue, Cambridge CB3 0FA, UK; E-Mails: ni245@cam.ac.uk (N.I.); MHo2@slb.com (M.Y.H.); mp380@cam.ac.uk (M.P.); qmm20@cam.ac.uk (M.M.Q.); 2 Institute of Nano Electronic Engineering (INEE), Universiti Malaysia Perlis (UniMAP), Lot 106, 108 & 110, Tingkat 1, Block A, Taman Pertiwi Indah, Jalan Kangar-Alor Setar, Seriab 01000 Kangar, Perlis, Malaysia; 3 Schlumberger Cambridge Research, High Cross, Madingley Road, Cambridge CB3 0EL, UK; 4 State Key Laboratory of Bioelectronics, School of Biological Science and Medical Engineering, Southeast University, Sipailou 2, Nanjing 210096, China; E-Mails: huaxu@seu.edu.cn (H.X.); gu@seu.edu.cn (Z.G.)

**Keywords:** explosives, functionalized nanoparticles, colorimetric, label-free, self-assembly film, aptamer

## Abstract

This proof-of-concept study proposes a novel sensing mechanism for selective and label-free detection of 2,4,6-trinitrotoluene (TNT). It is realized by surface chemistry functionalization of silica nanoparticles (NPs) with 3-aminopropyl-triethoxysilane (APTES). The primary amine anchored to the surface of the silica nanoparticles (SiO_2_-NH_2_) acts as a capturing probe for TNT target binding to form Meisenheimer amine–TNT complexes. A colorimetric change of the self-assembled (SAM) NP samples from the initial green of a SiO_2_-NH_2_ nanoparticle film towards red was observed after successful attachment of TNT, which was confirmed as a result of the increased separation between the nanoparticles. The shift in the peak wavelength of the reflected light normal to the film surface (*λ_peak_*) and the associated change of the peak width were measured, and a merit function taking into account their combined effect was proposed for the detection of TNT concentrations from 10^−12^ to 10^−4^ molar. The selectivity of our sensing approach is confirmed by using TNT-bound nanoparticles incubated in AptamerX, with 2,4-dinitrotoluene (DNT) and toluene used as control and baseline, respectively. Our results show the repeatable systematic color change with the TNT concentration and the possibility to develop a robust, easy-to-use, and low-cost TNT detection method for performing a sensitive, reliable, and semi-quantitative detection in a wide detection range.

## 1. Introduction

Sensing of trace explosives such as 2,4,6-trinitrotoluene (TNT) is a complex and challenging task due to the lack of inexpensive sensors with high selectivity and sensitivity [1], the lack of easily detectable signals, and wide selection of explosive compositions [2,3]. Furthermore, explosive-based terrorism has grown tremendously in recent years, causing enormous damage to public safety and environmental pollution because of the simplicity and variety of schemes by which these explosive-based weapons can be deployed [4,5,6]. Being the primarily used nitroaromatic explosives produced during military preparation of landmines [7,8,9,10,11,12], TNT is also one of the key sources of dangerous water contamination [13].

Many approaches have been explored for TNT detections. Current sensing methods of nitro-based explosives are gas and liquid chromatography [14], mass spectrometry [15], ion-mobility spectroscopy [16], enzymatic assays [17], and electrochemical detection [18]. A simple and label-free alternative is to use optical detection in association with designed colorimetric arrays of charge-transfer acceptor/donor complexes made of colloidal mesoporous nanoparticles based on specific color responses via donor–acceptor interactions between TNT and primary amines [19,20], but how to detect TNT in a wide range of concentrations using such a method remains to be investigated.

In this work, we report a simple and label-free colorimetric detection of different TNT concentrations ranging from 10^−12^ to 10^−4^ molar using the surface chemistry functionalization technique developed by Ho *et al.* [18]. The feasibility of this approach was demonstrated by the colorimetric change from green of amine-functionalized silica nanoparticle film towards red after successful attachment of TNT, with the shift of the peak wavelength (*λ_peak_*) of the reflected light normal to the film surface together with the peak broadening. Selective binding of negatively charged reporter aptamer (AptamerX) with TNT was used to confirm the selectivity of our approach in the same TNT concentration range of 10^−12^ to 10^−4^ molar, with 2,4-dinitrotoluene (DNT) and toluene as the control and baseline, respectively.

Our colorimetric sensing employs the assembly of colloidal mesoporous silica nanoparticles. The nanoparticles offer a large sensing surface area, availability to integrate functional groups, and capability to immobilize into an inorganic mesopores network with active molecules [21,22,23,24,25,26,27,28]. In addition, the mesoporous particles in suspension can stay suspended over a long period of time, making them ideal for surface coatings [29]. Some fluorogenic detection of nitroaromatic explosives using colloidal mesoporous nanoparticles has been reported, including luminescent colloidal oligo(tetraphenyl)silole nanoparticles in THF/H_2_O suspensions [30], nanoscopic-capped mesoporous hybrid materials or MCM-41 support [31], and silica mesoporous supports gated with tetrathiafulvalene derivatives [32].

To prepare the samples in use, we do not use those materials which require expensive dyes or dopants, such as conjugated polymers [33], fluorescent dye molecules [34], quantum dots [35], and hybrid assemblies of materials [36]. Instead, silica nanoparticle surfaces were firstly functionalized with an amine head group (–NH_2_) as capturing probes for the TNT target. This step helps us to fully utilize the primary –NH_2_ reaction sites with TNT. Electron-rich amine ligands and electron-deficient aromatic ring interaction produce an amine–TNT charge-transfer σ-adduct (Meisenheimer complex) [37,38] as a colored compound. Further literature study [39,40,41,42,43,44] suggests that this σ-adduct forms at the methyl group of TNT molecule; as a result, a negative charge resonates and is stabilized by three electron withdrawing nitro groups (-NO2) on the molecule. The immobilized TNT nanoparticles were incubated with a TNT-specific aptamer to produce TNT–aptamer complexes in the second step.

Aptamers have a specific three-dimensional shape that binds to a broad range of targets from single molecules to complex target mixtures or entire organisms with high selectivity and sensitivity [45]. Aptamer–target binding is a result of structure compatibility, electrostatic and van der Waals interactions, stacking of aromatic rings, and hydrogen bonds, or from a combination of these effects [46]. The TNT-specific aptamer used in this study was designed by Ho *et al.* [18] using *in vitro* selection or SELEX (systematic evolution of ligands by exponential enrichment) [45,46,47,48] technology. SELEX process involves a library of 10^14^ to 10^15^ random nucleotide sequences incubated directly with the target, allowing the randomized nucleotide pool to interact and adopt different conformations with the target. Higher affinity sequences from the eluted pool of nucleic acid species were then amplified using polymerase chain reaction (PCR) for deoxyribonucleic acid (DNA), or reverse transcription polymerase chain reaction or RT-PCR for ribonucleic acid (RNA) for several iterative cycles. Generally, aptamers with high specificity for the target require about 15–25 cycles of selection, influenced by the stringency of the selection conditions [48].

**Figure 1:**
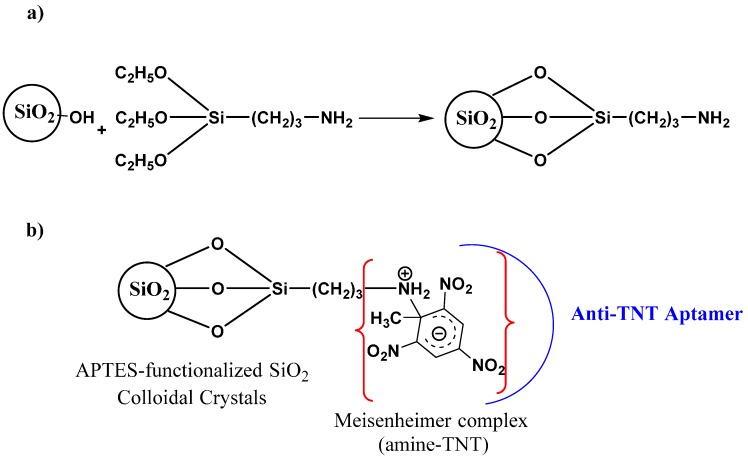
A schematic of functionalized silica nanoparticles (SiO_2_), (**a**) a capturing probe with terminal amine group (–NH_2_), and (**b**) a Meisenheimer amine–TNT complex interacting with a 100-base-pair aptamer reporter.

Functionalization of silica nanoparticles with a primary amine head group is shown in Figure 1a. The TNT target is then sandwiched between the anchored amine head group on silica nanoparticles and the aptamer, as illustrated in Figure 1b.

## 2. Experimental Section

### 2.1. Materials

Silica nanoparticles with a diameter size of 240 ± 10 nm, suspended in a stock concentration of 30% w/v in water, were purchased from Dongjian Biological (China); 18.2 MΩ-cm Millipore water, ammonium hydroxide solution (NH_4_OH) 28% NH_3_ in H_2_O, (≥99.99% trace metals basis); anhydrous ethanol (EtOH) (>99.9%); acetonitrile (anhydrous, 99.8%), cleaning solvent such as acetone and isopropyl alcohol (IPA); APTES (99%); *p*-benzoquinone for spectrophotometric detection of amines, ≥99.5% (HPLC); 10 mM HEPES + 150 mM NaCl; HEPES solution (1 M, pH 7.0–7.6); and sodium chloride (NaCl) were purchased from Sigma Aldrich. 2,4,6-Trinitrotoluene and 2,4-dinitrotoluene solutions (1000 μg/mL in acetonitrile) were obtained from the Defence Science and Technology Laboratory (DSTL); quartz substrate of 3 cm × 0.1 nm was purchased from Pi-KEM—Spectrosil B Polished Windows/Quartz with thickness of 1.25 mm and diameter of 24.6 ± 0.10 mm. 2 µM of negative charged aptamer (AptamerX) with sequence of 5′-g gat ccg ttg ata taa aat tcc aca tat cac ata ccg agc gcg cga cgt cgt ctc act gtc ctg ctg cct ccg cgt cat ggt tga ttg tgg tgt tgg ctc-3′ in 10 mM Phosphate buffer, pH 7, was prepared by using 20 µM aptamer and 10 mM HEPES + 150 mM NaCl.

### 2.2. Functionalization of Nanoparticles Surface

The silica (SiO_2_) nanoparticles suspension was prepared in anhydrous ethanol via solvent replacement method. First, water suspension was evaporated to dryness, followed by addition of ethanol using these parameters: 4000 rpm centrifugation speed for 5 min and sonication for 10 min, three times. The SiO_2_ nanoparticles suspended in ethanol was chemically functionalized in a solution of 18.2 MΩ-cm Millipore water, ammonium water (NH_4_OH), and pure ethanol. The mixture was stirred in a flask placed on a hotplate and 5 mM APTES was slowly stirred into the flask for 6 h at 60 °C. After 6 h of continuous stirring, the hotplate was turned off with further stirring for 30 min, and left overnight. The amine-functionalized SiO_2_ nanoparticles (SiO_2_-NH_2_) then went through a series of solvent replacement until the nanoparticles were fully dispersed in pure ethanol, giving a milky or hazy appearance.

### 2.3. Spectrophotometric Detection of Primary Amines

Spectrophotometric detection of amines was made by reacting 10^−4^ molar *p*-benzoquinone reagent with various concentrations of amines. The SiO_2_-NH_2_ suspension was diluted seven times and reacted with 10^−4^ molar *p*-benzoquinone. By using UV-NIR absorption spectra scanned from 190 nm to 1100 nm, it was estimated that 3.61 mM of primary amine was functionalized on the silica nanoparticle surfaces.

### 2.4. Explosives and Anti-TNT Aptamer Attachment

Attachment of TNT with the amine head group was done following the method performed by Ho *et al.* [18]. A series of TNT and DNT concentrations of 10^−12^, 10^−10^, 10^−8^, 10^−6^, and 10^−4^ molar were examined. DNT and toluene in acetonitrile were used as control and baseline, respectively. The acetonitrile solvent, in TNT and DNT, was left to dry in a fume cupboard for 2 h, and added with pure ethanol to the initial volume level of 50 µL. This step is crucial since acetonitrile is immiscible with water based-buffer of 10 mM HEPES + 150 mM NaCl. While waiting for the drying of acetonitrile, 5.54 µL of SiO_2_-NH_2_ nanoparticles suspended in ethanol was mixed with 200 µL of 10 mM HEPES + 150 mM NaCl for 2 h, which was expected to saturate all the available –NH_2_ reaction sites. Fifty microliters of various TNT concentrations in pure ethanol were then added to the saturated SiO_2_-NH_2_ nanoparticles suspension in 10 mM HEPES + 150 mM NaCl and incubated for an hour with minimal shaking using a vortex machine. The product of the reaction between SiO_2_-NH_2_ nanoparticles and TNT (SiO_2_-NH_3_-TNT) was produced after centrifugation at 2000 rpm for 90 s, which was followed by washing with pure ethanol three times. Twenty-five microliters of SiO_2_-NH_3_-TNT at various concentrations were incubated with 100 base pairs of 25 µL 2 µM anti-TNT AptamerX for 1 h. The product of the reaction between AptamerX and TNT samples was then centrifuged twice at 1000 rpm for 90 s, and washed with 10 mM HEPES + NaCl, before storage in 10 mM phosphate buffer at pH 7.

### 2.5. Self-Assembled Film Deposition

The functionalized nanoparticles were self-assembled on 3 cm × 0.1 cm quartz substrates, cut using a dicing machine. The substrates were cleaned with acetone and IPA for 15 min each in an ultrasonic bath and blow dried with a nitrogen gun before use. Fifty microliters of functionalized nanoparticles were deposited on a quartz substrate via solvent evaporation method in ambient temperature, for 48 h.

### 2.6. Characterization Methods

**High-resolution microscopy**: Olympus BX51 Microscope was used to capture the film surfaces with 10 × magnification lens with Q Imaging RETIGA-4000R High Sensitivity IEEE 1394 Firewire Digital CCD Camera and Image-PRO Plus software.

**Spectrometric measurement** was used to quantify the color band change, in terms of peak wavelength (*λ_peak_*) of the reflected light from the self-assembled films for sensing explosives, using Spectrophotometer Ocean Optics HR2000 + CG High Resolution Spectrometer for Biological and Chemical Application with measuring software SpectraSuite. Spectronic GENESYS 6 (UV-Visible Spectrophotometer) by Thermo Electron Corporation and Thermo Scientific™ VISIONlite™ software were used to measure the concentration of primary amines on silica nanoparticles.

**Scanning Electron Microscopy (SEM)** was used to examine the functionalized nanoparticle films. The films were first sputtered with gold (Au) at 55 mA for 15 s before measurement with a coating thickness of about 14 nanometers. SEM imaging analysis was done at a low accelerating voltage of 3 kV using Ziess Scanning Electron Microscopy, with SmartSEM imaging software.

**PeakFit v4.12 Automated Peak Separation Analysis Software** was used to measure the peak wavelength width and resolved multiple peak wavelengths.

## 3. Results and Discussion

**Figure 2:**
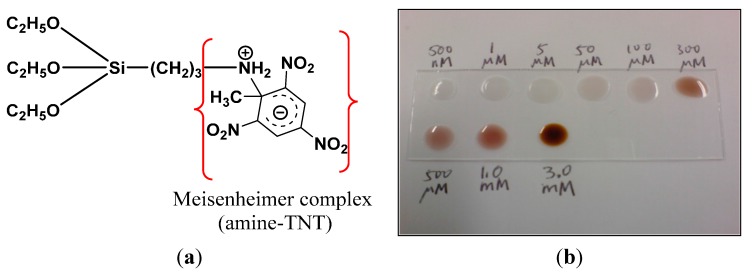
The Meisenheimer amine–TNT interaction demonstrates: (**a**) reaction between the amine group and TNT molecule and (**b**) a gradual change in color to the dark red of a TNT anion as the explosive concentration increases.

We first examined the interaction between TNT and amine groups by taking 8 μL of TNT at different concentrations and mixing with 8 μL of APTES, which also has a primary amine head group. A gradual change to a dark red color is observed in Figure 2 as the concentration of TNT increases. This is caused by the red-colored TNT anion [37,43]. The detection range by the naked eye is limited to 100 µM. On the other hand, using a high-resolution optical instrument, it is possible to detect a much lower range, as we show later in the paper.

**Figure 3:**
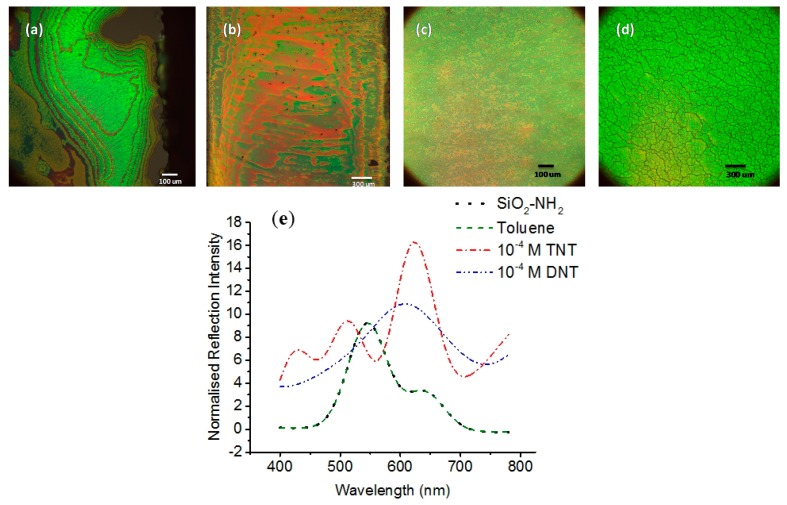
(**a**) SiO_2_-NH_2_ NP SAM film with bright green color, as observed under a high-resolution microscope; (**b**) 10^−4^ M TNT functionalized nanoparticles with strong color change and red areas for the attachment of TNT; (**c**) control sample of 10^−4^ M DNT functionalized nanoparticle film with very little color change; and (**d**) blank sample of toluene-functionalized nanoparticle film with no color change. The normalized reflected spectrum of SAM films (**a**)–(**d**) is represented in (**e**). The corresponding $\lambda_{peak}\;$ of film (**a**,**d**), (**b**), and (**c**) are 545 nm, 631 nm, and 607 nm, respectively.

To demonstrate the sensor sensitivity, detection of TNT at lower concentrations ranging from 10^−12^ to 10^−4^ molar was performed and the colorimetric change after TNT attachment on amine-functionalized silica nanoparticles was observed. For example, the self-assembled (SAM) film changes color from the luminescent green of SiO_2_-NH_2_ and SiO_2_-NH_2_-toluene (blank) nanoparticles towards red after the successful attachment of 10^−4^ molar TNT, as shown in Figure 3. The red TNT anion is caused by the strong acceptor/donor interaction between TNT and amino ligands, respectively [37,38,43], and proven to exhibit a large shift in the *λ_peak_* as compared to the DNT control.

SiO_2_ nanoparticles offer great advantages as a sensing material because of their large sensor surface area, label-free and intrinsic luminescence properties, and high specificity binding for organic molecules. Both the amine attachment on silica nanoparticle surfaces shown in Figure 3a and the baseline in Figure 3d correspond to a *λ_peak_* of 545 nm in wavelength. After the TNT binding illustrated in Figure 3b, the *λ_peak_* increases to 86 nm, whereas DNT-functionalized nanoparticles present a smaller shift of 62 nm but with a significant peak broadening, as displayed in Figure 3e.

Our approach to the colorimetric detection of TNT is to measure the average reflected light of the red areas that are uniform in terms of spectrum with 0.5% variation. The spectrophotometric measurement was done in air. As seen in Figure 3b, the red and green areas on the film correspond to the TNT-attached SiO_2_-NH_2_ and the SiO_2_-NH_2_ nanoparticles, respectively. The red areas observed are a result of strong acceptor–donor nitrogen interaction between the nitro group of TNT and the amine group. Incubation with a blank material such as toluene shows no change in the film color, as illustrated in Figure 3d. This indicates that toluene does not modify the structure of SiO_2_-NH_2_ nanoparticles, making it a perfect baseline for the spectrophotometric measurement from 400 nm to 800 nm. Further, the DNT-functionalized film seen in Figure 3c shows no obvious pattern, unlike the TNT film with its distinctive red areas. DNT and amine do not have such a strong interaction as TNT and the amine head group.

### 3.1. Colorimetric Measurement

Colorimetric change of the TNT-functionalized nanoparticle film was measured by means of reflected light normal to the film surface at 400 nm to 800 nm wavelength. A highly reflective silver mirror was used as the baseline and, due to different reflectivity of the films and mirror, a different integration time was applied. The normalized reflected intensity of the film was calculated based on the following expression:

$$\text{Normalized reflection intensity}=\frac{\left(\frac{\text{Film reflection intesity}}{M\text{irror reflection intensity}}\right)}{\left(\frac{\text{Film integration time}}{M\text{irror integration time}}\right)}\times 100\%$$

**Figure 4:**
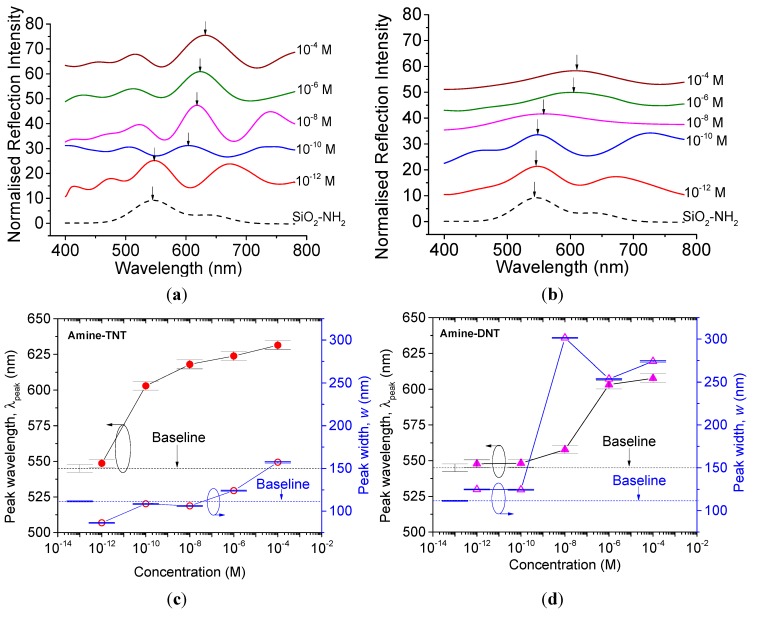
Normalized reflection intensities measured on (**a**) TNT- and (**b**) DNT-functionalized nanoparticle films with 15% offset for different compositions, and the corresponding peak wavelength, $\lambda_{peak}\;$with the associated peak widths, w of (**c**) TNT and (**d**) DNT samples. The baselines for peak shift and peak width are of the blank material, a toluene-functionalized nanoparticle film, with $\lambda_{peak,Baseline}\;$545 nm and$\;w_{B}\;$111.56 nm.

Figure 4a,b shows the measured spectrum of the TNT- and DNT-functionalized nanoparticle films, respectively. For clarity, the spectrums were offset by 15%. The peak wavelength axis in Figure 4c,d shows the corresponding $\lambda_{peak}\;$positions from Figure 4a,b, and their associated peak widths for all explosive concentrations. The sensitivity of the sensors is taken as the minimum TNT concentration in use, 10^−12^ molar, for which the reflection peak already shifted above the baseline $\lambda_{peak}\;$of 545 nm_._ The concentration range of the samples in this measurement is from 10^−12^ to 10^−4^ molar.

**Figure 5:**
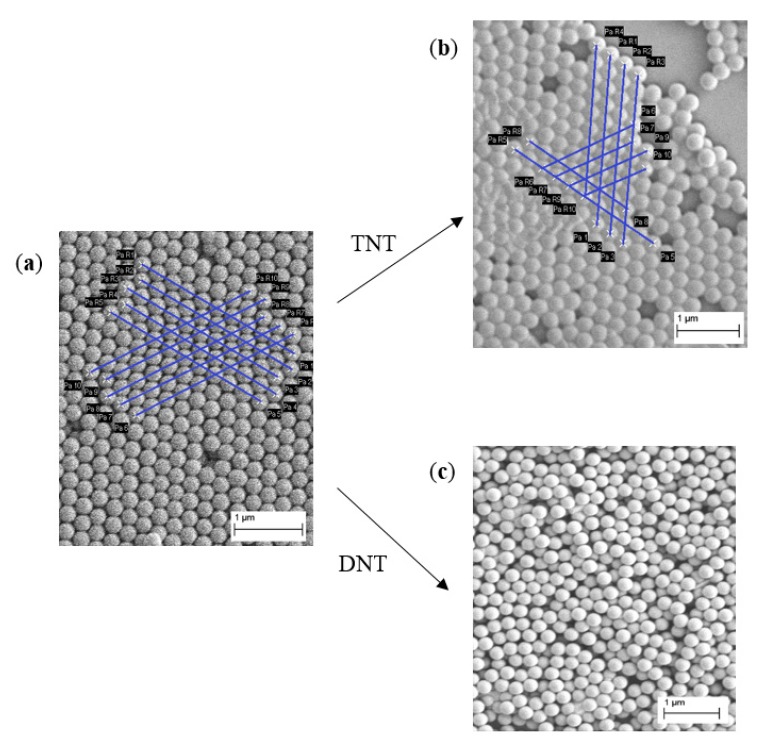
SEM images for the separation between nanoparticles (**a**) without and (**b**) with TNT binding, as measured at 257.7 nm (baseline) and 270.9 nm (for 10^−4^ M TNT), respectively; and (**c**) randomized nanoparticles in a DNT film due to incomplete reaction, which contributes to the peak broadening in such films.

As shown in Figure 4a,b, the peak wavelengths are shifted towards longer wavelengths. At the same time, the peak widths of DNT samples are much broader than those of TNT samples of the corresponding concentrations, as shown in Figure 4c,d. The shift in the peak wavelength $\lambda_{peak\;}$is the result of the change of separation between nanoparticles after functionalization, while peak broadening is attributed largely to the incomplete reaction and resulted randomization, as shown in Figure 5.

**Figure 6:**
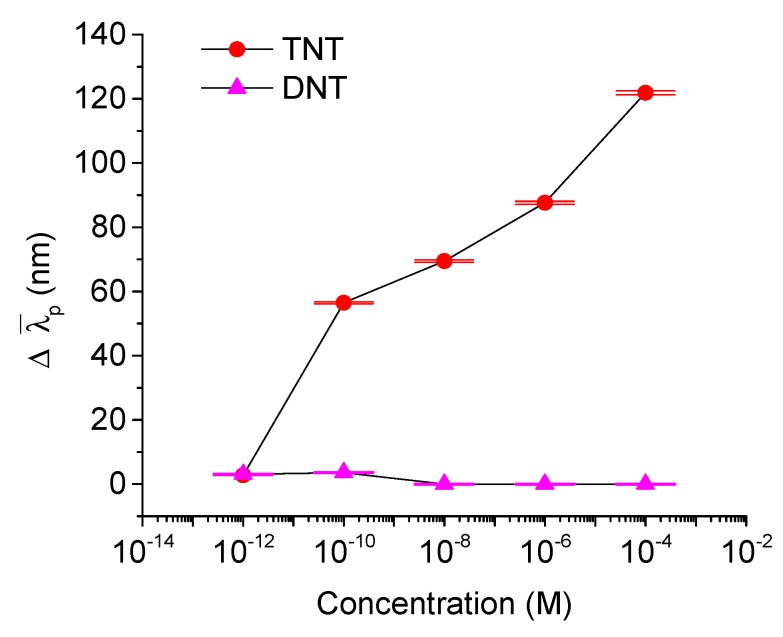
The weighted peak shifts ($∆{\bar{\lambda }}_{p}$) for TNT and DNT samples of different concentrations.

As a result, the latter can be used as a factor of discrimination as in Equation (2). We define the weighted peak shift$\;∆\bar{\lambda_{p}}$ as the peak shift$\;∆\left(\lambda_{p}\right)\;$multiplied by the normalized peak broadening$\;\left(\frac{w}{w_{B}}\right)\;$relative to the reference material:

(2) $$∆{\bar{\lambda }}_{p}=∆\lambda_{p}\;\times \left(\frac{w}{w_{B}}\right)\times \{\begin{array}{c} 1\;w<2\times w_{B}\;\\ 0\;w\geq 2\times w_{B} \end{array}$$

where the

$$∆\lambda_{p}\;=\lambda_{peak}\;-\lambda_{peak,Baseline}$$

w is the peak width of the measured explosive film, and$\;w_{B}\;$is the peak width of the reference material (toluene in this case). The factor of 2 is used to limit the range of intermolecular reaction so that it is not too far away from the starting point (reference material). By considering the peak broadening effect in Equation (2), we found out that not only can it provide a monotonic link with the concentration but also a nearly linear relationship with the$\;∆{\bar{\lambda }}_{p}\;$for TNT-functionalized nanoparticle films, as shown in Figure 6. Equation (2) considers the baseline of a toluene-functionalized nanoparticles film with $\lambda_{peak,Baseline\;}$of 545 nm and$\;w_{B}\;$of 111.56 nm. Both$\;∆{\bar{\lambda }}_{p}\;$for TNT and DNT samples of different concentrations are shown in Figure 6.

### 3.2. Sensing Selectivity

The selectivity of our colorimetric sensing can be confirmed through the selective binding between AptamerX and 2,4,6-trinitrotoluene, TNT [45,46,47,48], in comparison with the colorimetric response of the bindings between AptamerX and the control materials 2,4-dinitrotoluene, DNT, and blank toluene. We followed a two-step procedure [18] by fully utilizing the primary –NH_2_ reaction sites with TNT first, as reported in Section 3.1, and then incubating the immobilized TNT nanoparticles with aptamer for an hour to produce TNT–aptamer complexes. TNT, DNT of various concentrations, and toluene-functionalized nanoparticles (formed in Section 3.1) were incubated with 100 base pairs of 2 µM AptamerX. They were then self-assembled on quartz substrates for approximately 48 h. In this experiment, toluene-aptamer functionalized nanoparticle film was used as the baseline.

**Figure 7:**
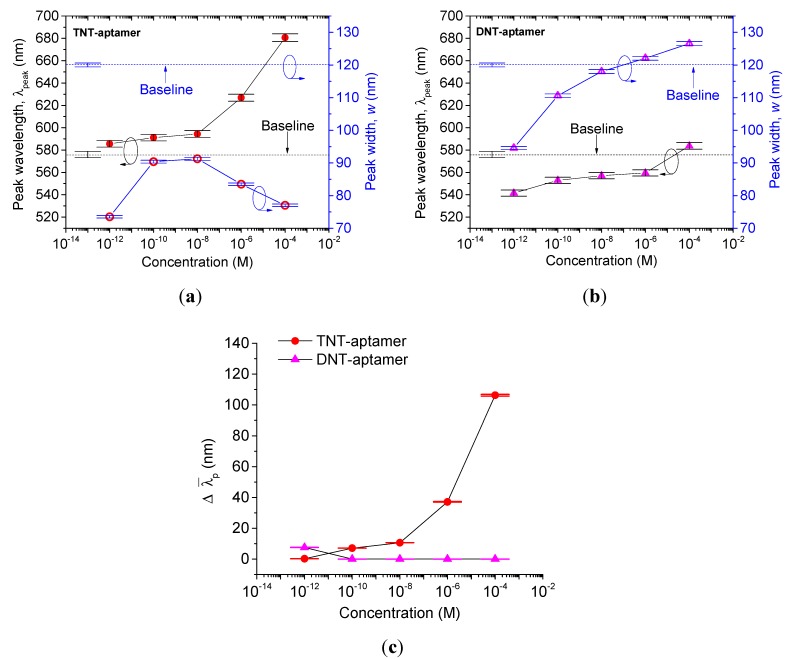
The corresponding peak wavelength, $\lambda_{peak}\;$with the associated peak widths, w of (**a**) TNT–aptamer, (**b**) DNT–aptamer functionalized nanoparticle films, and (**c**) the$\;∆{\bar{\lambda }}_{p}\;$for TNT–aptamer and DNT–aptamer films of different explosive concentrations. The baselines are of a toluene–aptamer film with $\lambda_{peak,Baseline\;}$576 nm and$\;w_{B}\;$of 120 nm.

In Figure 7a, the reflection peak of the TNT–aptamer complex shifted towards longer wavelengths as the aptamer was introduced to higher concentrations of TNT. Note that this result is independent of the first step of primary amine interaction with TNT. Thus the peak wavelength and peak width depend entirely on the arrangement of the TNT–aptamer functionalized nanoparticles in the SAM film that they form. The peak wavelengths and the associated peak widths of TNT–aptamer and DNT–aptamer functionalized nanoparticle films of different concentrations are shown in Figure 7a,b, respectively. The corresponding$\;∆{\bar{\lambda }}_{p}\;$is plotted in Figure 7c, showing high selectivity towards TNT–aptamer film. The$\;∆{\bar{\lambda }}_{p}\;$values of the TNT–aptamer samples are comparatively lower than those of the amine–TNT samples, because of the larger$\;w_{B\;}\;$of 120 nm.

## 4. Discussions and Conclusions

Large surface area of nanoparticles can lead to the high sensing sensitivity and stability of a self-assembled film. The colorimetric response examined in this work was focused on dried nanoparticle films, from which the reflected light was studied. We expect that the reflected light from the functionalized nanoparticles in liquid will behave differently, which may reveal more information regarding the interactions between them.

In conclusion, we demonstrated that films of self-assembled functionalized nanoparticles can be used for simple, selective, and label-free colorimetric sensing for a wide range of TNT concentrations from 10^−12^ to 10^−4^ molar. They show reliable color changes after successful binding of TNT. We showed that the redshift in the peak wavelength $\lambda_{peak\;}$is a result of the increase in the average separation between nanoparticles after functionalization and TNT attachment, while the peak broadening is largely attributed to the randomization of nanoparticles due to incomplete reaction. As a result, the latter can be used as a factor of discrimination. By considering the peak broadening effect, we obtained not only a monotonic but also a linear relationship with the logarithmic concentration of TNT for repeatable detections. This also allowed us to reliably distinguish the TNT-functionalized nanoparticles from the DNT control and toluene blank samples in the whole composition range we studied. Finally, an aptamer was used to confirm the good selectivity of the TNT functionalized nanoparticles. Consequently, it is reasonable to believe that our sensing approach has the potential to be further developed for robust, easy-to-use, and low-cost label-free TNT sensing applications.
